# Lateral lymph node metastasis in papillary thyroid cancer: Is there a difference between PTC and PTMC?

**DOI:** 10.1097/MD.0000000000037734

**Published:** 2024-04-26

**Authors:** Wenli Ma, Yehao Guo, Tebo Hua, Linlin Li, Tian Lv, Jiafeng Wang

**Affiliations:** a Graduate School of Bengbu Medical University, Bengbu, China; b Zhejiang Provincial People’s Hospital Bijie Hospital, Bijie, China; c Otolaryngology & Head and Neck Center, Cancer Center, Department of Head and Neck Surgery, Zhejiang Provincial People’s Hospital, Affiliated People’s Hospital, Hangzhou Medical College, Hangzhou, China; d Key Laboratory of Endocrine Gland Diseases of Zhejiang Province, Hangzhou, China; e Wenzhou Medical University, Wenzhou, China; f Department of Thyroid Breast Surgery, Ningbo Medical Centre Lihuili Hospital, Ningbo, China; g Hangzhou Normal University, Hangzhou, China.

**Keywords:** lateral lymph node metastasis, lateral neck lymph node dissection, N1b, papillary thyroid carcinoma, papillary thyroid microcarcinoma

## Abstract

Papillary thyroid carcinoma (PTC) and papillary thyroid microcarcinoma (PTMC) are generally characterized as less invasive forms of thyroid cancer with favorable prognosis. However, once lateral cervical lymph node metastasis takes place, the prognosis may be significantly impacted. The purpose of this study was to evaluate whether there is a difference in the pattern of lateral lymph node metastasis between PTC and PTMC. A retrospective analysis was performed for PTC and PTMC patients that underwent central area dissection and unilateral lateral neck lymph node dissection (II–V area) between January 2020 and December 2021. Compared with PTMC group, the PTC group exhibited higher incidence of capsule invasion, extrathyroid invasion and lymphatic vessel invasion. Both the number and rate of central lymph nodes metastasis were elevated in the PTC group. While the number of lateral cervical lymph node metastasis was higher, the metastasis rate did not demonstrate significant difference. No significant differences were identified in the lymph node metastasis patterns between the 2 groups. The determination of the extent of lateral neck lymph node dissection solely based on the tumor size may be unreliable, as PTC and PTMC showed no difference in the number and pattern of lateral neck metastasis. Additional clinical data are warranted to reinforce this conclusion. For patients categorized as unilateral, bilateral, or contralateral cervical lymph node metastasis (including level I, II, III, IV, or V) or retropharyngeal lymph node metastasis who require unilateral lateral neck dissection, the size of the primary tumor may not need to be a central consideration when assessing and deciding the extent of lateral neck dissection.

## 1. Introduction

In recent decades, there has been a noticeable increase in the incidence of thyroid cancer across diverse populations globally,^[1]^ and this trend as also been reflected in that of papillary thyroid cancer.^[2,3]^

Thyroid cancer is a common endocrine malignancy, of which papillary thyroid carcinoma (PTC) is the most common subtype, accounting for 80% of thyroid malignancies. PTC has become one of the most prevalent tumors in China,^[4]^ but it is less aggressive by nature and usually has a better prognosis, with a 5-year disease-specific survival rate of >98%.^[5]^ What remains to be addressed, is the common complication of lymph node metastasis.^[6]^ Papillary thyroid microcarcinoma (PTMC) refers to PTC whose maximum diameter is not larger than 1 cm.^[7]^ In recent years, developments in diagnosis and treatment methods along with patient awareness have contributed to detections of PTC or PTMC during early stages.^[1,2]^ Researchers like Feng et al^[8]^ have posited that distinctions may exist between PTC and PTMC, which is supported by studies demonstrating differences between PTC and PTMC concerning lymph node metastasis in the central area.^[9]^ Given that preoperative evaluation are commonly done with noninvasive ultrasound, the presence of cervical lymph node metastasis (CLNM) is harder to determine due to being more concealed anatomically. This brings significance to findings by Feng et al^[10]^ that suggested PTC has a stronger tendency of lymph node metastasis (CLNM) in the central region. Our study builds upon this information and gauges risks of CLNM by incorporates assessment through presence and extent of lateral cervical lymph node metastasis that is more easily detected, and may thus be more practical clinically.

Lateral cervical lymph node metastasis is one of the major factors affecting patient prognosis. While early lateral cervical lymph node metastasis may not significantly affect prognosis, it is considered a predictor for progression and recurrence.^[11]^ In addition, different treatment methods and protocols can produce different prognostic outcomes depending on the type of pathology and characteristics of the lateral cervical lymph node metastases (LNM).^[12,13]^ Therefore, this study aimed to investigate the differences in lateral cervical lymph node metastasis in PTC and PTMC to provide a reference for clinical treatment and prognostic evaluation involving potential CLNM.

## 2. Materials and methods

### 2.1. Patient selection

A retrospective study was conducted for pN1b PTC patients who simultaneously underwent total thyroidectomy, central LN dissection (CLND), and ipsilateral therapeutic lateral LN dissection (LLND) from January 2020 to November 2021 at Zhejiang Provincial People’s Hospital.

**Figure 1.**
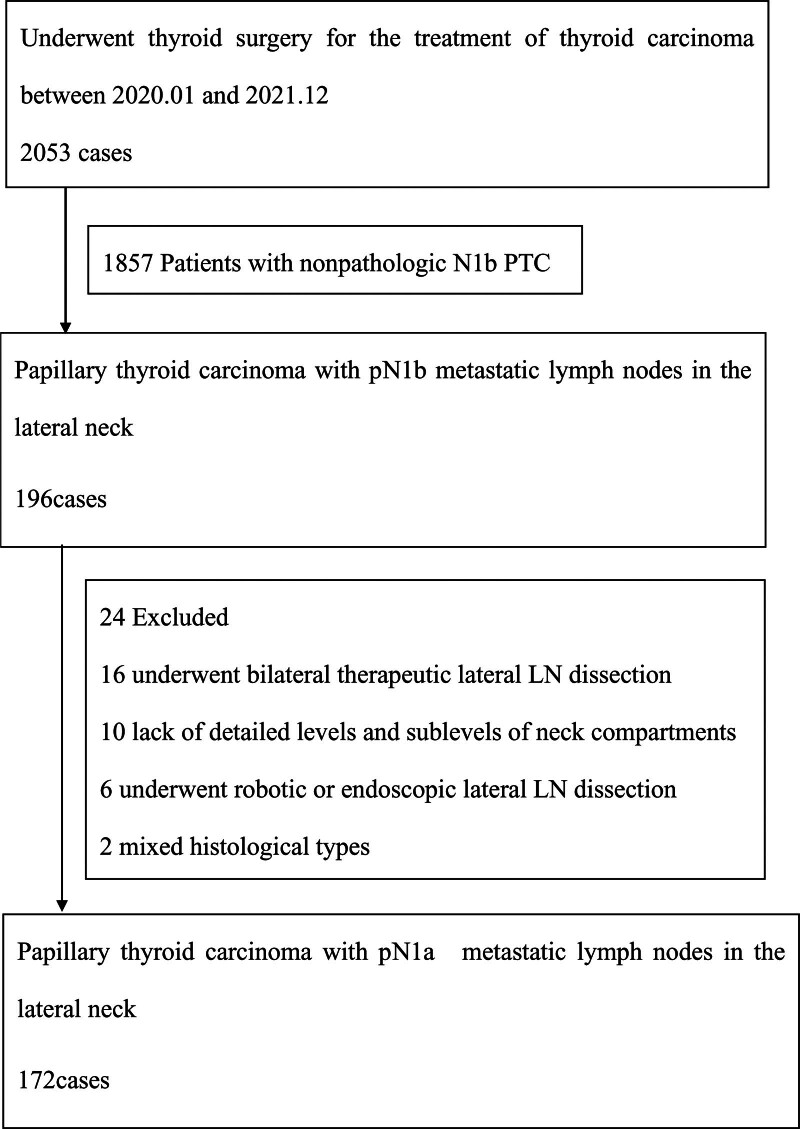
Flow diagram of literature search. LN = lymph node, PTC = papillary thyroid carcinoma.

The exclusion criteria were previous thyroid surgery, lack of detailed levels and sublevels of neck compartments, mixed histological types, underwent bilateral therapeutic lateral LN dissection, and underwent robotic or endoscopic lateral LN dissection. Detailed inclusion and exclusion criteria and the resulting number of patients after each selection step are shown in Figure 1. After exclusion, this study enrolled 172 patients with pN1b PTC. The patients were divided into 2 groups: the PTMC group whose maximum diameter of the primary tumor was ≤1 cm and the PTC group whose maximum diameter was larger than 1 cm. Data from PTMC and PTC groups were compared. The study was approved by the medical ethics review committee at Zhejiang Provincial People’s Hospital.

### 2.2. Surgical strategy

Physical examination, ultrasound, and enhanced computed tomography (if no contraindication) were routinely performed on all patients. Positive imaging findings should be addressed using US-guided fine needle aspiration biopsy or fast-frozen pathology to confirm lateral neck disease. All unilateral, bilateral, or contralateral cervical lymph node metastasis (including level I, II, III, IV, or V) or retropharyngeal lymph node metastasis (N1b) PTC patients received total thyroidectomy, CLND (level VI), and LLND (levels II-V). Classification of the neck nodes was according to the American Thyroid Association Consensus Review and Statement Regarding the Anatomy, Terminology, and Rationale for Lateral Neck Dissection in Differentiated Thyroid Cancer.

### 2.3. Data extraction and analysis

First, the following variables were analyzed between the PTMC and PTC groups: gender, age at diagnosis, maximum tumor diameter, maximum short diameter of metastatic lymph nodes, tumor location in the thyroid gland, multifocality, bilaterality, capsular invasion, extrathyroidal extension (ETE), lymphovascular invasion, thyroiditis, metastatic CLN count and ratio. Second, the LLNM pattern including metastatic LLN count and ratio, the distribution of LLNM in each level, and multilevel LLNM were also compared between the PTMC and PTC groups.

### 2.4. Statistical analysis

Statistical analysis was performed using SPSS Version 26.0 for Mac (IBM, USA). Quantitative data are presented as mean ± standard deviation. Between-group differences concerning categorical variables were assessed with Chi-squared test or Fisher exact test. Univariate and multivariate analysis was performed to identify variables associated with central lymph node metastasis. Between-group differences associated with *P* values <.05 were considered to be statistically significant.

## 3. Results

Thyroidectomy, central ventricular neck dissection (VI level), and selective unilateral neck dissection (II–V level) were performed. All postoperative pathology were of N1b PTC. The patients were divided into PTMC (primary tumor size ≤10 mm) group and PTC group, PTMC group (n = 67) and PTC group (n = 105). There were 35 females (52.9%) and 32 males (47.1%) in the PTMC group and 47 females (40.8%) and 58 males (49.2%) in the PTC group. The average age of PTMC group was 42.33 and 40.76 years old for PTC group. Compared with PTMC group, there were more thyroid capsule invasion, extraglandular invasion, and vascular lymphatic invasion in PTC group. The maximum short diameter of cervical metastatic lymph nodes (MS-CMLN [mm]) in PTC group was larger than that in PTMC group. The number of metastatic central lymph nodes (metastatic CLN count) and the ratio of metastatic central lymph nodes (metastatic CLN ratio) in PTC group were higher than those in PTMC group, that is, PTC group is more likely to have central lymph node metastasis than PTMC group, and once central lymph node metastasis occurs, the number of central lymph node metastasis in PTC group will be more than that in PTMC group.

There was no significant difference in multifocal, bilateral cervical lymph node metastasis, upper 1/3 gland involvement, and Hashimoto thyroiditis (Table 1). Among the above factors, thyroid capsule invasion, extraglandular invasion, and vascular lymphatic invasion were more frequent in PTC group (*P* < .001). The maximum short diameter of cervical metastatic lymph nodes (MS-CMLN [mm]) was larger (*P* < .01), the number of metastatic central lymphoid nodules (metastatic CLN count) and the ratio of metastatic central lymphoid nodules (metastatic CLN ratio) in PTC group were higher than those in PTMC group (*P* < .001), yielding significant statistical differences compared with PTC group.

**Table 1:** Clinicopathologic variables of 172 patients with PTC according to tumor size.

Variables	PTMC N = 67	PTC N = 105	*P* values	χ^2^/*t*
Gender (male)	32 (47.1%)	58 (49.2%)	.783	0.076
Age (yr)	42.33 ± 12.44	40.76 ± 14.51	.467	0.729
BMI	23.30 ± 3.38	24.71 ± 4.17	.022	2.314
Maximum tumor diameter (mm)	6.60 ± 2.59	20.21 ± 13.11	<.001	9.551
Bilaterality	25 (37.3%)	45 (42.9%)	.470	0.521
Multifocality	33 (49.3%)	54 (51.4%)	.781	0.077
Capsular invasion	25 (37.3%)	74 (70.5%)	<.001	18.413
Extrathyroidal extension	6 (9.0%)	41 (39.0%)	<.001	18.651
Lymphovascular invasion	8 (11.9%)	39 (37.1%)	<.001	13.082
Upper third lobe involvement	40 (59.7%)	57 (54.3%)	.485	0.488
Thyroiditis	18 (26.9%)	34 (32.4%)	.442	0.590
MS-CMLN (mm)	7.07 ± 2.86	8.73 ± 5.20	.018	2.389
Positive CLN metastasis	52 (77.6%)	92 (87.6%)	.083	3.005
Metastatic CLN count	3.13 ± 3.46	5.62 ± 5.61	.001	3.251
Metastatic CLN ratio	0.39 ± 0.34	0.57 ± 0.34	.001	3.308

BMI = body mass index, CLN = central lymph nodes, MS-CMLN = maximum short diameter of cervical metastatic lymph nodes, PTC = papillary thyroid carcinoma, PTMC = papillary thyroid microcarcinoma.

**Figure 2.**
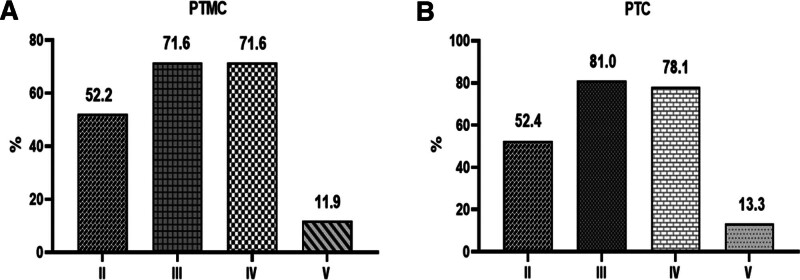
The pattern of lateral lymph node metastasis. PTC = papillary thyroid carcinoma, PTMC = papillary thyroid microcarcinoma.

We further summarized the diffusion pattern of lateral lymph node metastasis. The II–V area was involved in 35 cases (52.2%), 48 cases (71.6%), 48 cases (71.6%), and 8 cases (11.9%) in the PTMC group, and 55 cases (52.4%), 85 cases (81.0%), 82 cases (78.1%), and 14 cases (13.3%) in the PTC group, respectively (Fig. 2). There was no significant difference in the proportion of cervical lymph nodes between PTMC and PTC groups (*P* = .985, .155, .337, and .790 for II, III, IV, and V segments, respectively). There was some statistical difference between PTMC and PTC in the metastatic LLN count (*P* < .035), but there was no significant difference in the metastatic LLN ratio. This suggests the probability of lateral cervical lymph node metastasis in PTMC and PTC is the same, but once lateral cervical lymph node metastasis occurs, the number of metastatic lateral cervical lymph nodes in PTC is more than that in PTMC. There was no significant difference between cervical levels II, III, IV, and V (Table 2).

**Table 2:** The pattern of lateral lymph node metastasis in 172 patients with PTC according to tumor size.

Variables	PTMC N = 67	PTC N = 105	*P* values	χ^2^/*t*
Metastatic LLN count	4.40 ± 3.14	5.91 ± 5.26	.035	2.120
Metastatic LLN ratio	0.14 ± 0.10	0.16 ± 0.11	.225	1.22
Lever II metastasis	35 (52.2%)	55 (52.4%)	.985	0.000
Lever III metastasis	48 (71.6%)	85 (81.0%)	.155	2.022
Lever IV metastasis	48 (71.6%)	82 (78.1%)	.337	0.923
Lever V metastasis	8 (11.9%)	14 (13.3%)	.790	0.071

LLN = lateral lymph nodes, PTC = papillary thyroid carcinoma, PTMC = papillary thyroid microcarcinoma.

**Figure 3.**
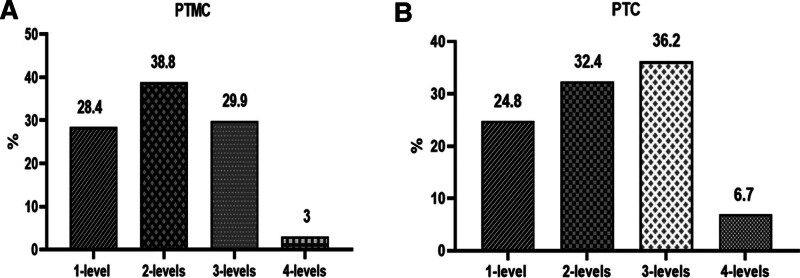
The number of affected levels of lateral lymph node metastasis. PTC = papillary thyroid carcinoma, PTMC = papillary thyroid microcarcinoma.

There were 19 (28.4%), 26 (38.8%), 20 (29.9%), and 2 cases (3.0%) involved 2 to 4 cervical lymph node zones in PTMC group, and 26 cases (24.8%), 34 cases (32.4%), 38 cases (36.2%), and 7 cases (6.7%) in PTC group, respectively (Fig. 3). There was no significant difference in the number of lateral cervical lymph nodes involved in metastatic lymph nodes between PTMC and PTC groups (*P* = .985, .155, .337, and .790, respectively), that is, once lateral cervical lymph node metastasis occurred, the regions of cervical lymph nodes involved by PTMC and PTC were the same (Table 3).

**Table 3:** The number of affected levels of lateral lymph node metastasis in 172 patients with PTC according to tumor size.

Number of affected levels	PTMC N = 67	PTC N = 105	*P* values	χ^2^
1-level	19 (28.4%)	26 (24.8%)	.601	0.274
2-levels	26 (38.8%)	34 (32.4%)	.389	0.743
3-levels	20 (29.9%)	38 (36.2%)	.391	0.736
4-levels	2 (3.0%)	7 (6.7%)	.485*	—

PTC = papillary thyroid carcinoma, PTMC = papillary thyroid microcarcinoma.

* The χ^2^ value could not be calculated.

We further analyzed the number of lymph nodes dissected, and found that the number of lymph nodes dissected in the central, lateral neck and total in the PTMC group was less than that in the PTC group, and the difference was statistically significant (*P* = .000, .007, .001) (Table 4).

**Table 4:** Comparison of the number of dissected lymph nodes in 172 PTC patients.

Variables	PTMC N = 67	PTC N = 105	*P* values	*t*
Dissected CLN count	7.73 ± 4.169	9.33 ± 6.693	.000	1.753
Dissected LLN count	31.18 ± 10.505	36.45 ± 19.149	.007	2.061
Dissected TLN count	38.90 ± 11.510	45.74 ± 23.713	.001	2.202

CLN = central lymph nodes, PTC = papillary thyroid carcinoma, PTMC = papillary thyroid microcarcinoma, TLN, total lymph nodes.

## 4. Discussion

There were differences between PTMC and PTC groups concerning thyroid capsule invasion, extraglandular invasion, the maximum short diameter of lymphatic vessels and cervical metastatic lymph nodes (MS-CMLN [mm]), the number of metastatic central lymph nodes (metastatic CLN) and the ratio of metastatic CLN. However, there was no significant difference in the number and mode of multi-focus, involving the upper lobe, bilateral cervical lymph node metastasis, and lateral cervical lymph node metastasis between the 2 groups.

Most PTMC behave in a relatively nonaggressive fashion, with a 15-year specific survival rate of 99% and a recurrence rate of 5%.^[14]^ However, some PTMC exhibit aggressive behavior at initial diagnosis, such as ETE and LLNM.^[15]^ For example, in our study, 72.6% of patients had multilevel LLNM. Lymph node metastases are a critical prognostic indicator in PTC. Both CLNM and LLNM increase the risk of recurrence, with LLNM additionally elevating the risk of distant metastasis and thus higher mortality.^[16]^ The occurrence of LLNM significantly raises the rate of postoperative complications.^[17]^ Thus, for patients requiring lateral cervical lymph node dissection in N1b PTMC, the relationship between functional protection and tumor safety should be fully evaluated to select the appropriate scope of surgical dissection.

Since the removal of lymph nodes in area V may lead to complications associated with paraspinal nerve injury, such as “shoulder syndrome”: drooping of the shoulder joint and inability to raise the arm more than 90°,^[18]^ such procedure may yield negative impacts on the patient’s social activities and quality of life. Therefore, whether dissection for level V should be routinely performed remains controversial.^[19,20]^ Kim et al^[18]^ reviewed the data of 646 patients with N1b papillary thyroid cancer and found that there was no statistically significant difference in the recurrence rate between patients undergoing selective neck dissection (levels II—IV) and modified radical neck dissection encompassing (levels II—V) surgery. Yang et al object to routine V-region lymph node dissection in patients with N1bPTMC citing a relatively low incidence of V-level metastasis and recurrence.^[21–23]^ Although the cited incidence is consistent with the results obtained in this study, the risk of global local recurrence rate is significantly increased when there are 3 levels of metastasis present where 1 includes level V metastasis. Thus, when metastasis transpires at level V, even if metastasis is only detected in 1 or 2 levels, it leads to a negative impact on the local recurrence rate.^[21]^ Therefore, accurately assessing the involvement of V level in lateral neck is paramount. This assessment underpins our principle of meticulously cleaning level V.

Arora et al^[24]^ previously compared PTMC with PTC finding no significant difference in multifocal tumor, ETE, lymphovascular invasion, and LN metastasis between the 2 groups. However, this study did reveal distinctions in extraglandular invasion between the 2 groups. This discrepancy may be attributed to the fact that our patient was classified as N1b and underwent total thyroidectomy, CLND, and ipsilateral therapeutic LLND. Consequently, our patient group might be characterized as being at a more advanced stage.

Recent active monitoring cohort studies have demonstrated that the median doubling time of PTC metastatic lymph nodes is 2.2 years, with a median growth rate of 1.4 mm/yr.^[25,26]^ This implies that the discovery of a lesion could indicate that it has been growing for an extended period. Additionally, lateral neck lymph node metastases are more likely to be detected preoperatively than smaller lesions. The higher ETE rate in the PTC group is also self-evident, as larger tumors have increased opportunities to invade the thyroid gland. In this study, although the PTC group required a more extensive lymph node clearance than the PTMC group in both central and lateral neck regions, possibly due to larger tumor sizes in the PTC group, which are a known risk factor for lymph node metastasis. There was no significant difference in the number or mode of lateral neck lymph node metastasis between the 2 groups. Lateral LN metastasis in both groups most frequently involved levels III and IV, which is consistent with previous reports.^[19]^ Zhu et al^[27]^ found that gender, maximum tumor diameter, and lesion location significantly influenced LLNM. Similarly, Feng et al^[28]^ identified tumor size, number, and lesion location as independent predictors of LLNM, while Zhuo et al^[29]^ highlighted gender, tumor size, tumor shape, vascularity of lymph nodes, and lymph node location as independent risk factors for LLNM. Wang et al^[30]^ concluded that multiplicity was an independent risk factor for both central lymph node metastasis (CLNM) and LLNM. In their study, Liu et al^[31]^ identified microscopic mETE, multifocality, serum thyroid-stimulating hormone levels, CLNM, and tumor size as risk factors for LLNM.

Our results show that although the larger primary tumor is associated with higher T stage and higher number of metastatic lymph nodes in the central area, it does not affect the number and pattern of lateral cervical lymph node metastasis. This implies that when surgeons decide on the type of lateral neck dissection, tumor size alone should not be used as an index to exclude tumor metastasis. Therefore, the treatment of PTMC with lateral cervical lymph node metastasis is no different from that of PTC with lateral lymph node metastasis larger than 1 cm, since the choice of selective and modified radical lateral lymph node dissection should not be guided solely by the size of the primary tumor.

However, our research is not without limitations. First, the study is a single-center retrospective study in which the number of participants is moderate, which may hinder the generalizability of our findings. Second, the number of PTMC cases is less than that of PTC, potentially leading to statistical biases. Finally, the study only involved the clearance of lymph nodes with confirmed metastasis or lymph nodes deemed suspicious preoperatively. We did not dissect some patients with contralateral lymph node metastasis who were not evaluated, or were evaluated before the operation. As a result, some occult lymph node metastases may have been overlooked. A more robust dataset and additional research will be necessary to address these challenges.

## Author contributions

**Conceptualization:** Tian Lv, Jiafeng Wang.

**Data curation:** Wenli Ma, Yehao Guo, Linlin Li, Jiafeng Wang.

**Formal analysis:** Tebo Hua.

**Investigation:** Tebo Hua.

**Writing—original draft:** Wenli Ma, Yehao Guo, Jiafeng Wang.

**Writing—review & editing:** Wenli Ma, Tian Lv, Jiafeng Wang.
